# Supplementary figures and images for: Wolbachia endosymbionts subvert the endoplasmic reticulum to acquire host membranes without triggering ER stress
Source: PLoS Negl Trop Dis. 2019 Mar 20;13(3):e0007218. doi: 10.1371/journal.pntd.0007218 (PMC6426186; doi:10.1371/journal.pntd.0007218)

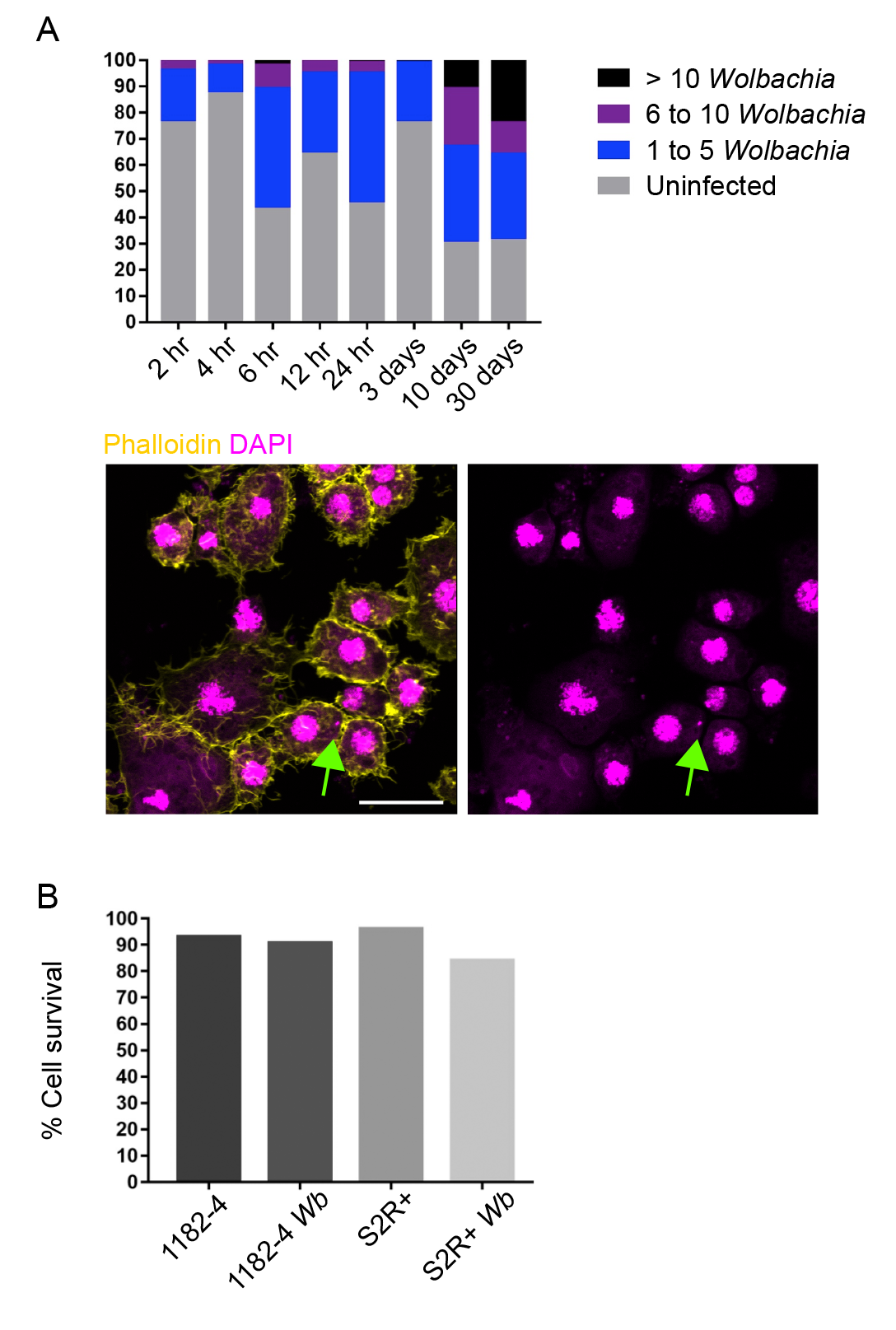

Supplement: S1 Fig — (A) Infection dynamics of 1182–4 cells challenged with purified wMel Wolbachia. Scoring of intracellular Wb was performed on confocal images of fixed cells at the various time points represented on the graph, with a phalloidin staining -yellow- to visualize the cortical actin in order to count the number of intracellular Wb only, per individual cells. Wb are detected as DAPI bright cytoplasmic foci(-magenta-, i.e. green arrow pointing at a single bacterium at an early time point). Scale bar = 10 μm, n = 100 cells per time point, counted in randomly acquired images per coverslip. (B) Cell survival established with Trypan blue. Analyses were performed 24 hr-post medium change. (TIF) [file pntd.0007218.s001.tif]

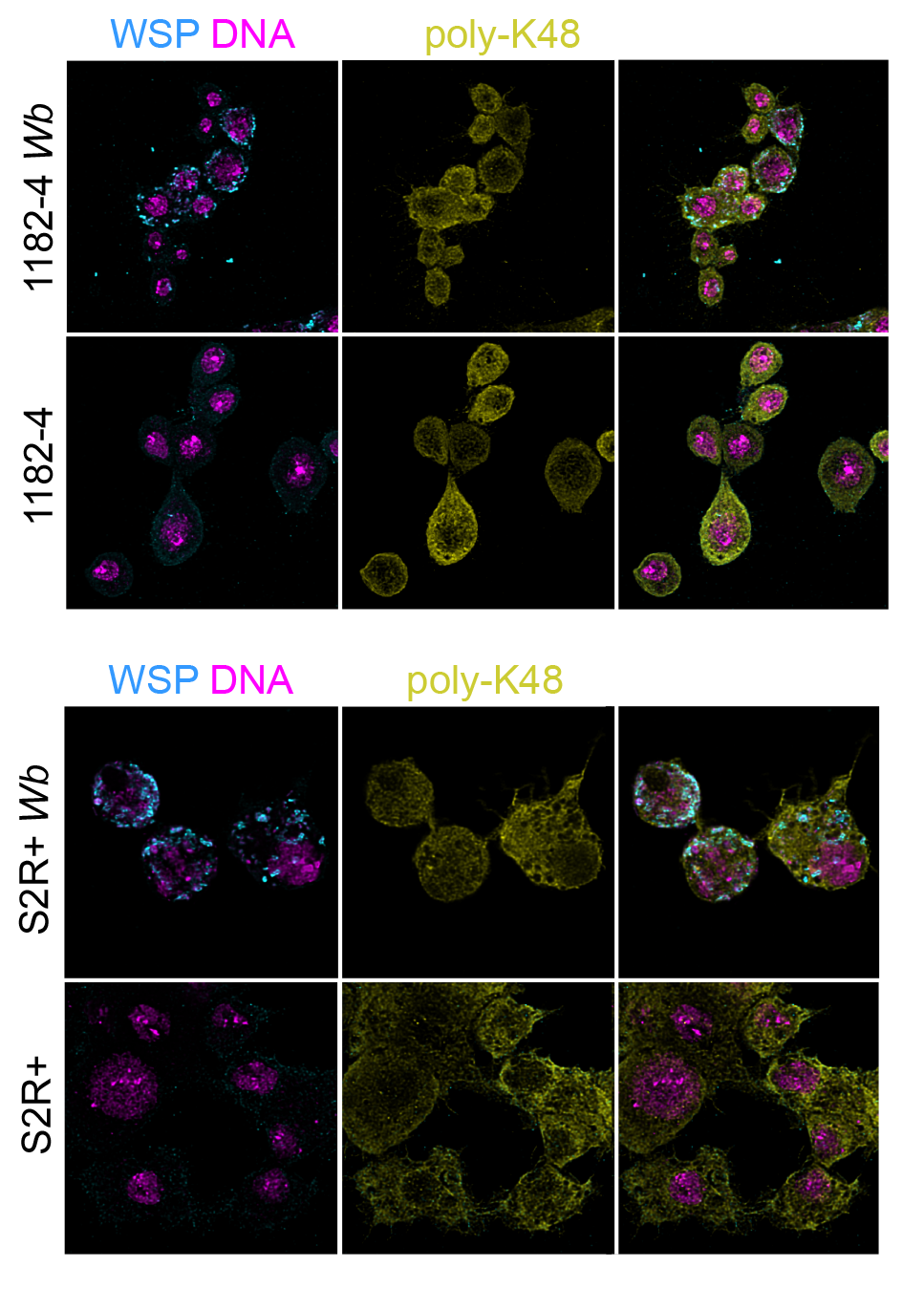

Supplement: S2 Fig — Confocal acquisitions of the infected and non-infected 1182–4 and S2R+ cell lines stained with WSP -magenta- and an anti- K48-linkage polyubiquitin -yellow-. (TIF) [file pntd.0007218.s002.tif]
