# Supplementary material for: Wolbachia endosymbionts subvert the endoplasmic reticulum to acquire host membranes without triggering ER stress
Source: PLoS Negl Trop Dis. 2019 Mar 20;13(3):e0007218. doi: 10.1371/journal.pntd.0007218 (PMC6426186; doi:10.1371/journal.pntd.0007218)
Supplement: S1 Table — (DOCX) [file pntd.0007218.s006.docx]

| **Gene in *Drosophila melanogaster*** | **Ortholog gene in mammals** | **Flybase annotation** | **Forward primer** | **Reverse primer** |
| --- | --- | --- | --- | --- |
| actin42A | actin | CG12051 | GCGTCGGTCAATTCAATCTT | AAGCTGCAACCTCTTCGTCA |
| gapdh2 | gapdh | CG8893 | TTTCTCAGCCATCACAGTCG | CGATGCGACCAAATCCAT |
| crc | atf4 | CG8669 | CCGCTGATTACCAGCTCAAT | GCAGATGCTGCGTGTGTAAC |
| atf6 | atf6 | CG3136 | GAATGCGATTCCCTAAAAGCT | GCAATGCTTTGCCTTTGATG |
| ire1 | ire1 | CG4583 | GAGATCACAGCGAACGACAA | GGATAATTCGGCTGTCCTCA |
| gcn2 | perk | CG1609 | AAAACCTGCCAAGTCACCAC | CAGAGTCCTCCATGTTGGGT |
| pek | perk | CG2087 | GGCTCGAACACTGCGTAGAT | CGTCCACTGATTACGGGTTT |
| xbp1 | xbp | CG9415 | TGGGAGGAGAAAGTGCAAAG | CGAGTCCAGCTTGTGGTTCT |
| CG1317 | doa10 | CG1317 | GCTTCGGCTATGATCTTTGG | GTGTTTGGGTCTGCTCGAAG |
| sip3 | hrd1 | CG1937 | TTATGATACCACCCCACTTCG | CGGAATCCAGCATTAGGTTT |
| hrd3 | sel1L | CG10221 | CTTCGACGGACTTCGAGACT | ATTGCTGCTCAGAGGCTTTC |
| der-1 | derlin-1 | CG10908 | GGGCATATTCGTGGGACAT | CTAGGTGGTGCTCTGCTCT |
| der-2 | derlin-2 | CG14899 | CGGCAGTTCTACCTGGAGAT | CTGATGCCTATTGTGCCAAA |
| ubc7 | ube2g2 | CG4443 | GAGCGTGGAGAAGATTTTGC | TTACGCCGGTAAACCAAGAG |
| plap | doa1 | CG5105 | GAGATTGGTGGCGTCAAGA | ACATCGCCTACCAAGTTCCA |
| ter94 | p97 | CG2331 | GATTACTAGCGCCCACTTCG | ACCAGATCCTGAGGTGTTGC |
